# Auditory processing from the perspective of auditory electrophysiological assessment

**DOI:** 10.1590/2317-1782/e20240332en

**Published:** 2026-02-02

**Authors:** Pedro de Lemos Menezes, Kelly Cristina Lira de Andrade, Ana Figueiredo Frizzo, Danielle Cavalcante Ferreira, Carlos Henrique Alves Batista, Eliane Schochat, Liliane Desgualdo

**Affiliations:** 1 Programa Associado de Pós-graduação em Fonoaudiologia – PPGFON, Universidade Estadual de Ciências da Saúde de Alagoas – UNCISAL - Maceió (AL), Brasil.; 2 Programa de Pós-graduação em Ciências da Saúde e Comunicação Humana, Departamento de Fonoaudiologia, Faculdade de Filosofia e Ciências, Universidade Estadual Paulista – UNESP - Marília (SP) Brasil.; 3 Faculdade de Medicina, Universidade de São Paulo – USP - São Paulo (SP), Brasil.; 4 Programa de Pós-graduação em Distúrbios da Comunicação Humana, Escola Paulista de Medicina, Universidade Federal de São Paulo – UNIFESP - São Paulo (SP), Brasil.

**Keywords:** Audiology, Auditory Evoked Potentials, Auditory Cortex, Speech Perception, Auditory Processing

## Abstract

**Purpose:**

To describe sound processing in the auditory system based on auditory evoked potentials.

**Research strategies:**

A literature review was conducted on auditory processing from the perspective of electrophysiological auditory assessment, considering both classical and current studies in the field.

**Selection criteria:**

Studies addressing auditory evoked potentials and their relationship with sound encoding, decoding, discrimination, perception, and semantic congruence processes were included.

**Data analysis:**

Data were analyzed in a descriptive and critical manner, integrating information on different auditory evoked potentials and their respective roles in auditory processing.

**Results:**

The auditory system organizes and encodes acoustic features, such as frequency, intensity, and temporal modulations, transforming them into neural representations interpreted by the cortex. Auditory evoked potentials provide information on encoding, decoding, discrimination, perception, and semantic congruence processes. The frequency-following response evaluates the accuracy of neural encoding of sounds, especially speech; cortical auditory evoked potentials reflect advanced processes of encoding, decoding, and discrimination; and the N400 is associated with semantic congruence, elucidating cognitive auditory processing.

**Conclusion:**

Auditory evoked potentials are important tools for evaluating auditory processing, contributing to the diagnosis of disorders and to the monitoring of auditory performance across different populations.

## INTRODUCTION

The auditory system is uniquely organized to extract behaviorally relevant information from complex acoustic environments, employing strategies that set it apart from other sensory systems^(1)^. Sounds such as human speech, music, and animal vocalizations contain acoustic information distributed across multiple frequencies and timescales ranging from a few milliseconds to several seconds. At the peripheral level, the auditory system encodes signals with diverse features - intensity, frequency, formants, amplitude modulation, frequency modulation, and sound-level dynamics – which are progressively processed by successive stations along the ascending auditory pathway, culminating in neural representations in the central auditory system, including the auditory cortex^(1,2)^.

**Figure 1:**
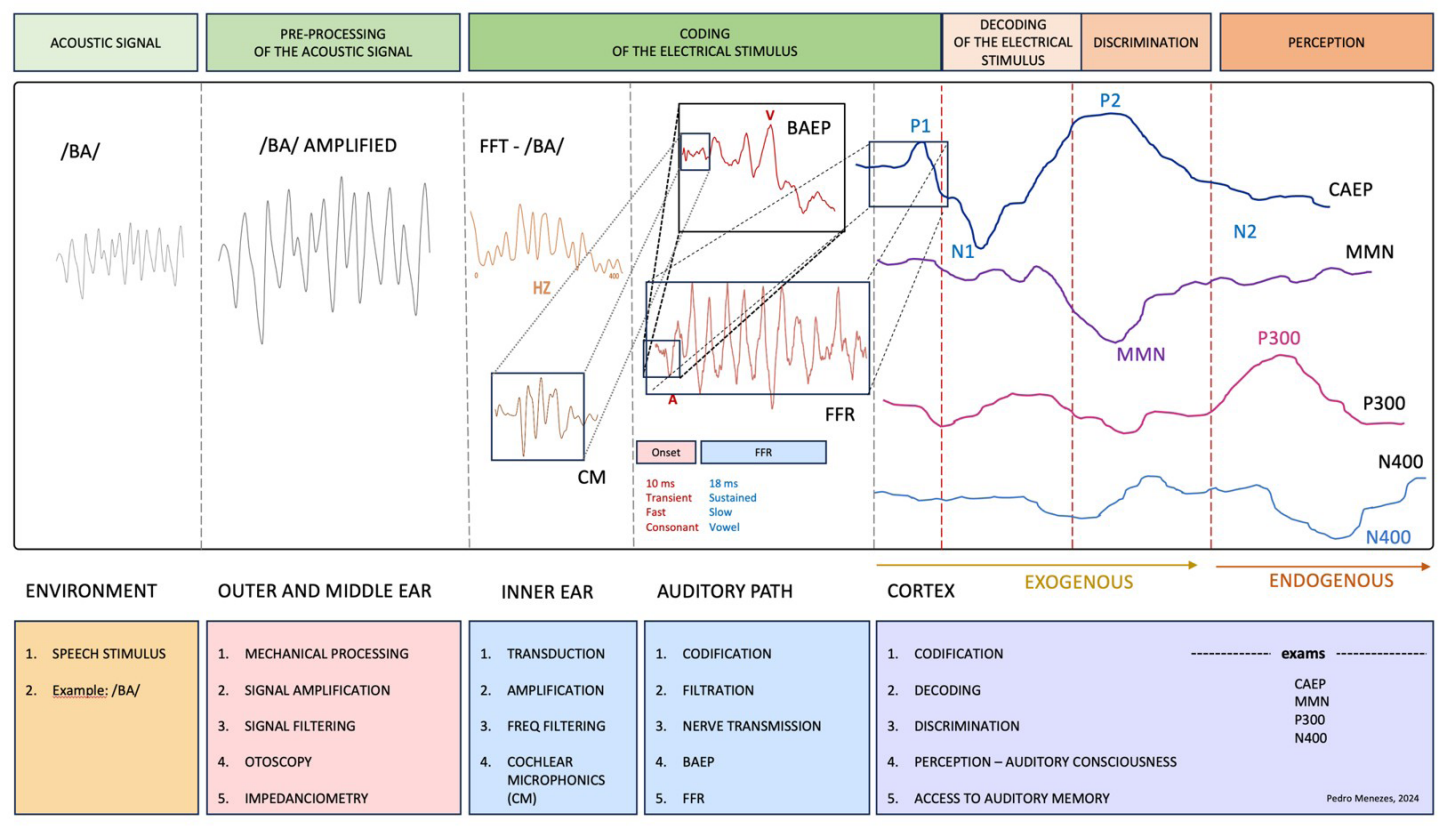
The figure illustrates the auditory processing model, highlighting its stages, from the acoustic characteristics of sound, its preprocessing, the electrical stimulus encoding, its decoding, discrimination, and perception. Furthermore, it shows the electrophysiological tests at each stage, and what happens to the sound from its production in the environment to its interpretation in the cortex . Free figure license: The auditory processing and its relationship with auditory evoked potentials © 2024 by Pedro de Lemos Menezes is licensed under CC BY-NC-SA 4.0

In this context, Auditory Evoked Potentials (AEP) emerge as an indispensable tool for mapping auditory processing. AEPs consist of bioelectrical recordings that reflect neural activity in response to sound stimuli and enable the assessment of different stages of this processing, from transduction in the cochlea to sound interpretation by the brain^(3)^, as shown in Figure 1. Specific techniques, such as the analysis of cortical and cognitive potentials, reveal responses to standardized and deviant stimuli, allowing the identification of subtle changes in central sound processing - a sensitive marker of auditory disorders^(4)^.

The clinical relevance of AEPs is demonstrated by their broad applicability, including early detection of hearing disorders, intraoperative monitoring, and screening in vulnerable populations, such as newborns and individuals with neurological conditions. In parallel, their scientific importance lies in their ability to elucidate the neurophysiological mechanisms underlying sound perception, contributing to the development of theoretical models that integrate the various levels of auditory processing^(5)^.

Thus, the objective of this review is to describe sound processing in the auditory system from the perspective of AEP, presenting a model that represents the complete sequence of steps - from the encoding of acoustic signal characteristics to their perception in the auditory cortex - and highlighting the clinical and scientific implications of this method for the diagnosis and rehabilitation of auditory disorders. Furthermore, it aims to present a model that represents all of this processing, from the observation of acoustic signal characteristics to their perception in the auditory cortex, in the form of a figure and with a free use license.

## METHOD

### Acoustics and hearing

Sound is defined as a vibratory phenomenon arising from air pressure variations capable of producing auditory perception. Sound must be within pressure and frequency variations compatible with the physiological characteristics of the human ear to be perceived. Sound pressure levels in the speech area are concentrated between 40 and 65 dB HL, while the highest-energy frequencies are between 400 and 4000 Hz^(6)^.

The frequency range capable of stimulating the human ear ranges from 20 to 20,000 Hz, but it varies with age. The lowest sound pressure level capable of promoting auditory perception has, on average, an intensity of 0 dB HL. The minimum detectable sound pressure, however, varies according to frequency, with a reference value of 20 μPa. The discomfort threshold for this same sound is 120 dB HL, and the pain threshold is 140 dB HL. Depending on the frequency, the same sound pressure may or may not be perceived by the auditory system^(6)^.

Sound stimuli reaching the ear are conducted via air and bone conduction to the peripheral auditory system (external ear, middle ear, inner ear, and vestibulocochlear nerve), where sound is captured and mechanical energy is transduced into nerve impulses. Nerve fibers exiting the cochlea (inner ear) reach the central auditory system (auditory pathways of the brainstem, thalamus, and auditory cortex), where sound is understood^(7)^.

Sound waves reaching the outer ear travel through the external auditory canal and cause vibrations in the eardrum. The ossicles of the middle ear transmit these vibrations to the oval window of the cochlea. In the cochlea, specifically in the Corti organ, the outer hair cells (OHCs), in contact with the tectorial membrane, move tonotopically, depending on the sound frequency^(8,9)^.

Movement of the OHCs amplifies the movement of the basilar membrane at their respective vibrational frequency. This amplification causes contact between the inner hair cells (IHCs) and the tectorial membrane, ultimately leading to mechanoelectrical transduction. Stimulation of the IHC cycles opens potassium ion channels, causing cell depolarization, neurotransmitter release, and the encoding of acoustic information into electrical impulses that reach the central nervous system via acoustic nerve, a branch of the vestibulocochlear nerve^(8,9)^.

### Sound processing in the auditory system

The central nervous system plays a fundamental role in several functions, such as memory, attention, language, and others. The auditory system shares neuroanatomical structures and processes with other systems. The way the central auditory system processes verbal and nonverbal sounds changes. Therefore, different types of stimuli can generate different response patterns^(10)^.

Speech understanding in the auditory system is a complex process that involves internal and external factors, such as the acoustic features of the sound and the presence of noise^(11)^. Speech is a complex acoustic signal characterized by a variety of properties, including the presence of harmonics, amplitude variations, and rapid changes in the frequency spectrum^(12)^. Therefore, the individual must be able to detect rapidly changing sound patterns^(13)^.

Speech understanding depends on the integrity of the structures and connections responsible for encoding sounds, that is, representing their temporal and spectral characteristics. The transduction of sound into electrical impulses in the cochlea and the processing of sound along the auditory pathways are essential for sound perception. Therefore, any impairment in these processes can impair speech understanding^(14)^.

This ability to understand auditory information is defined as central auditory processing (CAP), which refers to the efficiency with which the central nervous system uses auditory information. This requires a set of auditory skills and abilities responsible for the ability to locate, discriminate, recognize, store, and understand auditory information^(15)^. Alterations in CAP are caused by problems in the central auditory system. These changes can occur independently of cognitive and language impairments.

### Sound processing assessment

A variety of behavioral tests assess different auditory processing skills, including auditory discrimination, temporal processing, dichotic listening, low-redundancy speech recognition, and binaural interaction^(16)^.

In addition to basic audiological assessments, such as pure-tone and speech audiometry, acoustic immittance measures and otoacoustic emissions, electrophysiological tests can provide valuable information about the auditory pathway and its processing up to the auditory cortex^(16)^.

The American Academy of Audiology (AAA) recommends the use of AEPs to assess CAP, as they reflect fundamental auditory processing mechanisms such as encoding, decoding, discrimination, auditory awareness, and auditory memory access. Furthermore, they document the influence of maturation and intervention on CAP, and may be especially useful for intra-subject comparisons^(15)^.

When recording AEPs in response to the syllable /ba/ (Figure 1), for instance, it is possible to assess how the auditory system processes its physical-acoustic features, including the detection of changes in frequency, waveform, and sound duration. Such analyses contribute to a better understanding of the neural encoding of speech and can provide important insights about auditory function in different clinical contexts^(17)^.

### Cochlear microphonic

The cochlear microphonic (CM) is a potential generated primarily from the OHCs of the cochlea. The CM corresponds to an electrical activity that occurs prior to the synapses between the hair cells and the auditory nerve, and can be observed preceding wave I in the brainstem auditory evoked potential (BAEP) recording. Its absence is consistent with impaired function of these cells^(18)^.

The CM can be recorded through electrocochleography or BAEP, and it is necessary to invert the polarities of the acoustic stimulus to verify the inversion of the recording to confirm the CM in the BAEP^(18)^. Figure 1 shows the CM recording preceding wave I of the BAEP, highlighting its contribution to sound encoding.

This initial response is crucial, as it establishes the foundation for subsequent stages of auditory processing. By preserving the fidelity of the acoustic signal, the CM ensures that essential information, such as frequency, intensity, and timing, is accurately transmitted to following neural structures. This characteristic makes cochlear microphonics a valuable tool in both research and clinical practice, enabling early detection of hearing dysfunctions and contributing to the development of diagnostic and therapeutic strategies^(19)^.

### Brainstem auditory evoked potential

The BAEP is a short-latency Auditory Brainstem Response (ABR) that occurs within the first 10 milliseconds (ms) after the presentation of an acoustic stimulus and originates from the auditory nerve and auditory pathways in the brainstem, structures involved in sound encoding (Figure 1). The BAEP allows for objective assessment of individuals' responses at different intensities and is widely used to assess the integrity of auditory pathways to the brainstem and estimate electrophysiological hearing threshold. Its analysis consists of identifying three main wave peaks (I, III, and V), as well as tracing reproducibility, absolute latencies, amplitudes, interpeak intervals, and interaural wave difference^(20,21)^.

In BAEP, brief stimuli are commonly used, such as click, tone burst and chirp. Peaks I, III, and V are the most frequently analyzed in clinical practice as they have greater stability and amplitude, originating, respectively, in the distal portion of the auditory nerve, the cochlear nuclei, and the lateral lemniscus. However, the brief stimuli used in BAEP have a simple acoustic pattern that differs from environmental sounds, such as speech sounds, making this ABR limited for assessing brainstem behavior, especially when considering the processing of speech sounds in these structures^(20-22)^.

Finally, BAEPs capture initial sound encoding by recording, with high temporal precision, the electrical responses generated by the auditory nerve and brainstem pathways in the first 10 ms after stimulation, reflecting the neural synchronization essential for discriminating minimum temporal intervals and differentiating frequencies. This ability to record the peaks of waves I, III, and V, which represent distinct milestones in neural transmission, allows to assess how the auditory system processes and preserves the temporal and spectral characteristics of the acoustic signal, fundamental for the perception of complex sounds, such as speech, discussed in more detail below. Thus, BAEPs not only verify the structural integrity of the initial auditory pathways but also offer a valuable window into the study of temporal and frequency processing mechanisms, serving as an important tool both in clinical practice and in research on hearing disorders^(23)^.

### Frequency following response

The Frequency Following Response (FFR) is a noninvasive index of the fidelity of sound encoding in the brain and is used to study the integrity, plasticity, and behavioral relevance of neural sound encoding (Figure 1)^(24)^. This AEP is performed with speech stimuli, with the syllable /da/ used most frequently, which distinguishes it from other AEPs by reflecting the neural processing of the acoustic features of a sound and the speech encoding capacity^(24,25)^.

The FFR can be interpreted in the time domain, in which the response peaks identified in the waveform are: V, A, C, D, E, F and O. These waves have as probable generating sites the rostral brainstem, more specifically the lateral lemniscus and the inferior colliculus, in addition to the primary cortex^(26)^. In addition, by applying a Fast Fourier Transform (FFT), it is possible to perform a frequency-domain analysis, including components such as the fundamental frequency (F_0_), the first formant (F_1_), and the higher harmonics (HAs). The FFR responses are generated predominantly in the auditory midbrain, a center of both afferent and efferent activity, and therefore reflect multiple influences from the peripheral auditory pathway and the central nervous system^(24)^.

Thus, the FFR can be included in audiological testing and plays an important role in cross-checking. Its promising clinical results demonstrate potential for helping individuals of different ages, from infants to the elderly, with diverse needs, such as learning disabilities, attention deficit hyperactivity disorder (ADHD), and auditory processing disorder^(27)^.

Finally, the FFR excels in the continuous and accurate analysis of acoustic features essential to speech perception, as its ability to capture harmonics and formants provides a detailed neural representation that reflects the spectral nuances of the sound stimulus. This fidelity in signal preservation allows the identification of subtle variations in F0 and formant structure, critical elements for distinguishing phonemes and understanding prosody. Studies indicate that impaired neural encoding of these components may be associated with difficulties in speech perception, especially in noisy environments, reinforcing the relevance of the FFR in investigating the subcortical mechanisms of acoustic integration^(28,29)^. Thus, the FFR not only complements traditional audiological assessment but also represents a promising tool for improving diagnostic and therapeutic strategies in auditory processing disorders.

### Binaural Interaction component

The Binaural Interaction Component (BIC) is an electrophysiological measure that assesses the interaction between the auditory pathways of both cerebral hemispheres. Although it can be assessed at different auditory evoked potentials, it is frequently used to assess short-latency and middle-latency auditory electrophysiological responses. It is obtained by subtracting the responses evoked by monaural stimuli from the response evoked by binaural stimuli, reflecting the efficiency of sound information integration in the central auditory pathways^(30)^.The BIC can be calculated from the following generic equation:


$$\begin{array}{c} BIC=\;Response_{binaural}-\\ \left(Response_{left\;monaural}+Response_{right\;monaural}\right) \end{array}$$ (1)

This measure provides information about the integrity of neural connections responsible for spatial hearing and signal processing in complex environments, such as those with background noise^(31)^.

Behavioral binaural processing, on the other hand, refers to the ability to combine and distinguish sounds that reach both ears, and is fundamental for skills such as sound localization, auditory depth perception and speech understanding in noisy environments^(30)^.

Studies demonstrate a significant correlation between BIC and behavioral measures of binaural processing, such as masking level difference (MLD) and dichotic digit recognition^(32,33)^. This indicates that binaural interaction ability, as captured by BIC, is closely associated with behavioral performance in auditory tasks. Furthermore, both measures are equally influenced by variables such as age and auditory symmetry^(30-33)^. In particular, older individuals may present a reduced BIC and worse performance on behavioral tests, which may be indicative of central auditory processing deficits^(31)^.

### Cortical auditory evoked potential

The Cortical Auditory Evoked Potential (CAEP) is a long-latency AEP that occurs between 50 and 300 ms and is represented by the P1-N1-P2 complex that reflects the sound processing underlying auditory encoding, decoding, and discrimination abilities (Figure 1). Decoding corresponds to the interpretation of the temporal and spectral characteristics of a sound captured at encoding, while discrimination corresponds to the ability to detect, recognize, and distinguish differences between sounds^(34,35)^.

The P1 component originates from thalamic projections and the primary auditory cortex and is related to auditory encoding^(36)^. The N1 component stems from activations of the primary auditory cortex in the lateral temporal gyrus and is also influenced by the lateral temporal lobe, motor cortex, and frontal premotor cortex. This component is associated with attention and auditory decoding^(37)^. The P2 component, related to auditory discrimination, results from the joint activation of the primary auditory cortex and higher cortical areas, such as the supratemporal, frontal, and parietal regions. This complex activation allows the analysis of the acoustic and temporal characteristics of the stimulus^(36)^.

Thus, the progression of the CAEP components - P1, N1, and P2 - evidences the refined pathway of sound processing toward the cortex, reflecting successive stages of integration and interpretation of complex acoustic information. While P1 marks the initial encoding of signals, N1 represents an intermediate phase in which temporal and spectral aspects are integrated, and P2 denotes the more elaborate interpretation of acoustic elements, such as harmonics and formants, essential for speech perception. This functional sequence not only highlights the maturation and plasticity of the central auditory system but also emphasizes the CAEP clinical relevance in identifying and monitoring auditory processing disorders^(38,39)^.

CAEP responses do not depend on the active participation of the individual being examined; that is, they are an exogenous response that can be elicited by a variety of acoustic stimuli, from pure tones to speech stimuli. The use of speech stimuli, such as the syllable /ba/ represented in Figure 1, allows us to assess the auditory system's ability to process and discriminate the acoustic complexities of these stimuli^(34-37)^.

The CAEP significantly contributes to the CAP assessment, demonstrating its usefulness in the objective assessment of sound processing up to the level of the central auditory nervous system. In young children, the P1 component is best visualized on the tracing and has been considered a neurophysiological biomarker of auditory development, as its latency and amplitude decrease with age^(34,39)^.

### Mismatch negativity

The Mismatch Negativity (MMN) is a long-latency AEP that occurs around 100 to 350 ms. Its likely sites of generation are the supratemporal plane of the auditory cortex, posterior lateral temporal cortex, right frontal gyrus, and contributions from the thalamus. Its response is obtained from the CAEP tracings using the following equation:


$$Tracing_{MMN}=Tracing_{Deviant}-\;Tracing_{Standard}$$ (2)

The N1 and P2 wave components of the CAEP are directly related to the MMN. The N100, often used as a reference, precedes the MMN, which manifests as a negative trough after it. The P200, in turn, associated with more complex cognitive processes, can provide additional information about the detection of the change detected by the MMN^(40)^.

The MMN is an indicator of the early stage of auditory processing and can provide objective measures of auditory discrimination and automatic processing of sound perception as it is recorded even in the absence of conscious attention to the sound stimulus (Figure 1). This suggests that the brain is continually processing auditory information at a preconscious level^(40)^.

Finally, the MMN reflects the neural system's ability to anticipate and detect discrepancies in acoustic information, serving as a sensitive marker of the integrity and plasticity of auditory pathways. This ability is closely linked to central auditory processing, where complex neural circuits integrate and interpret sound signals to construct meaningful perceptions. Therefore, the MMN not only demonstrates the functionality of auditory pathways but also the brain's ability to discriminate sounds stored in memory^(4,41)^.

### Cognitive auditory evoked potential

The cognitive auditory evoked potential, or P300, is considered a long-latency AEP generated from responses of the auditory cortex, frontal lobe, hippocampus, and sensory systems. It is an endogenous AEP, that is, it is generated voluntarily, as it requires the individual to perform a task while the acoustic stimuli are presented^(35,42)^.

This AEP exhibits a positive peak elicited by the identification of a rare stimulus among a series of frequent ones (oddball paradigm), during the performance of a specific discrimination task. The P300 component appears at approximately 300 ms and represents the largest positive peak following the N1-P2 complex (Figure 1).

Thus, the P300, as an endogenous AEP generated by cognitive responses of the auditory system, is closely connected to central auditory processing, as it reflects the integration of acoustic information with higher cognitive functions, such as attention and decision-making. The brain's ability to identify rare stimuli reflects the efficiency of the neural networks involved in auditory discrimination and sequential information processing, with active participation of the auditory cortical, parietal regions, and hippocampus. This capacity for cognitive modulation of auditory responses reinforces the importance of the P300 in assessing the interaction between central auditory perception processes and cognitive systems, offering valuable insights into the integrity of central auditory processing and its implications for attention and auditory memory^(5,43)^.

Therefore, based on P300 responses, it is possible to assess skills of cognition, attention, discrimination, memory, decision-making and sequential processing of auditory information^(34,35,42)^.

### N400

The N400 is a long-latency negative cognitive potential elicited by a semantically incongruent or unexpected word presented among other words with semantic connections. The N400 component is a negative peak found at approximately 400 ms responsible for capturing information about psycholinguistic aspects that are not assessed in any other behavioral and/or auditory assessment or approach^(43)^.

Thus, this AEP is considered a neurophysiological marker of sound/word discrimination and their semantic congruence relationship with a linguistic stimulus. The generation of the N400 component requires auditory attention and awareness, and is capable of reflecting understanding based on sensory processing and association of meaning^(44)^.

## CONCLUSION

The proposed model, based on the analysis of AEPs, offers an integrated and functional view of auditory processing, outlining a coherent narrative that encompasses the capture of initial acoustic characteristics to the cortical interpretation of sound stimuli. Each AEP discussed - from cochlear microphonic, BAEP, and FFR, which demonstrate the encoding and fidelity of signals at neural input level, to the BIC, CAEP, MMN, P300, and N400, which reflect the integration, encoding, decoding, discrimination, and cognitive response to stimuli - contributes to understanding the successive stages of sound processing. This comprehensive approach not only allows for a broader understanding of the diagnosis and monitoring of hearing disorders but also supports targeted therapeutic interventions, demonstrating the practical and clinical relevance of the model in assessing the integrity and plasticity of auditory pathways, and the overall function of the auditory system.
