# Supplementary material for: Auditory processing from the perspective of auditory electrophysiological assessment
Source: Codas. 2026 Feb 2;38(1):e20240332. doi: 10.1590/2317-1782/e20240332en (PMC12978338; doi:10.1590/2317-1782/e20240332en)
Supplement: Translation

# Translation: O processamento auditivo sob a perspectiva da avaliação eletrofisiológica auditiva

**DOI:** 10.1590/2317-1782/e20240332pt

**Authors:** Pedro de Lemos Menezes, Kelly Cristina Lira de Andrade, Ana Figueiredo Frizzo, Danielle Cavalcante Ferreira, Carlos Henrique Alves Batista, Eliane Schochat, Liliane Desgualdo

**Affiliations:** 1 Programa Associado de Pós-graduação em Fonoaudiologia – PPGFON, Universidade Estadual de Ciências da Saúde de Alagoas – UNCISAL - Maceió (AL), Brasil.; 2 Programa de Pós-graduação em Ciências da Saúde e Comunicação Humana, Departamento de Fonoaudiologia, Faculdade de Filosofia e Ciências, Universidade Estadual Paulista – UNESP - Marília (SP) Brasil.; 3 Faculdade de Medicina, Universidade de São Paulo – USP - São Paulo (SP), Brasil.; 4 Programa de Pós-graduação em Distúrbios da Comunicação Humana, Escola Paulista de Medicina, Universidade Federal de São Paulo – UNIFESP - São Paulo (SP), Brasil.

**Keywords:** Audiologia, Potenciais Evocados Auditivos, Córtex Auditivo, Percepção da Fala, Processamento Auditivo

## Abstract

**Objetivo:**

Descrever o processamento do som no sistema auditivo a partir dos potenciais evocados auditivos.

**Estratégia de pesquisa:**

Foi realizada uma revisão da literatura sobre o processamento auditivo sob a perspectiva da avaliação eletrofisiológica auditiva, considerando estudos clássicos e atuais da área.

**Critérios de seleção:**

Foram incluídos estudos que abordaram os potenciais evocados auditivos e sua relação com os processos de codificação, decodificação, discriminação, percepção e congruência semântica do som.

**Análise dos dados:**

Os dados foram analisados de forma descritiva e crítica, integrando informações sobre diferentes potenciais evocados auditivos e seus respectivos papéis no processamento auditivo.

**Resultados:**

O sistema auditivo organiza e codifica características acústicas, como frequência, intensidade e modulações temporais, transformando-as em representações neurais interpretadas pelo córtex. Os potenciais evocados auditivos fornecem informações sobre os processos de codificação, decodificação, discriminação, percepção e congruência semântica. O Frequency Following Response avalia a precisão da codificação neural dos sons, especialmente da fala; os potenciais evocados auditivos corticais refletem processos avançados de codificação, decodificação e discriminação; e o N400 está associado à congruência semântica, elucidando o processamento auditivo cognitivo.

**Conclusão:**

Os potenciais evocados auditivos são ferramentas importantes para a avaliação do processamento auditivo, contribuindo para o diagnóstico de transtornos e para o monitoramento do desempenho auditivo em diferentes populações.

## INTRODUÇÃO

O sistema auditivo possui uma organização única que reflete a necessidade de extrair informações comportamentais relevantes de um ambiente acústico complexo, utilizando estratégias distintas daquelas empregadas por outros sistemas sensoriais^(1)^. Sons como a fala humana, a música e as vocalizações animais contêm informações acústicas distribuídas em múltiplas frequências e em escalas de tempo que variam de alguns milissegundos a vários segundos. No nível periférico, o sistema auditivo codifica sinais com características diversas – intensidade, frequência, formantes, modulação de amplitude, modulação de frequência e dinâmica de nível sonoro – que são progressivamente processadas por estações sucessivas ao longo da via auditiva ascendente, culminando em representações neurais no sistema auditivo central, incluindo o córtex auditivo^(1,2)^.

Nesse contexto, os Potenciais Evocados Auditivos (PEA) emergem como uma ferramenta indispensável para mapear o processamento auditivo. Os PEA são registros bioelétricos que refletem a atividade neural em resposta a estímulos sonoros e possibilitam a avaliação de diferentes etapas desse processamento, desde a transdução na cóclea até a interpretação do som pelo cérebro^(3)^, como representado na Figura 1. Técnicas específicas, como a análise dos potenciais corticais e cognitivos evidenciam respostas a estímulos padronizados e desviantes, permitindo identificar alterações sutis no processamento central do som – um marcador sensível de disfunções auditivas^(4)^.

**Figura 1:** A figura ilustra o modelo de processamento auditivo, destacando suas etapas, desde as características acústicas do som, seu pré-processamento, a codificação do estímulo elétrico, a sua decodificação, discriminação e percepção. Além disso, mostra os exames eletrofisiológicos, em cada etapa, e o que acontece com o som desde sua produção no ambiente até sua interpretação no córtex. Licença gratuita da figura: The auditory processing and its relationship with auditory evoked potentials © 2024 by Pedro de Lemos Menezes is licensed under CC BY-NC-SA 4.0

A relevância clínica dos PEA é comprovada por sua ampla aplicabilidade, incluindo a detecção precoce de distúrbios auditivos, o monitoramento intraoperatório e a triagem em populações vulneráveis, como recém-nascidos e indivíduos com condições neurológicas. Paralelamente, sua importância científica reside na capacidade de elucidar os mecanismos neurofisiológicos subjacentes à percepção sonora, contribuindo para o desenvolvimento de modelos teóricos que integram os diversos níveis do processamento auditivo^(5)^.

Dessa forma, o objetivo desta revisão é descrever o processamento do som no sistema auditivo sob a perspectiva dos PEA, apresentando um modelo que represente a sequência completa de etapas – desde a codificação das características do sinal acústico até sua percepção no córtex auditivo – e evidenciando as implicações clínicas e científicas desse método para o diagnóstico e a reabilitação de distúrbios auditivos. Além disso, visa apresentar um modelo que represente todo o referido processamento, desde a observação das características do sinal acústico até sua percepção no córtex auditivo, na forma de figura e com licença de uso gratuita.

## MÉTODO

### Acústica e audição

O som é definido como um fenômeno vibratório oriundo de variações de pressão no ar capaz de produzir percepção auditiva. O som deve estar dentro das variações de pressão e frequência compatíveis com as características fisiológicas da orelha humana para ser percebido. Os níveis de pressão sonora da área da fala estão concentrados entre 40 e 65 dBNA, enquanto as frequências de maior energia estão entre 400 e 4000 Hz^(6)^.

A faixa de frequência capaz de estimular a orelha humana é de 20 a 20000 Hz, mas varia com o avanço da idade. O nível de pressão sonora mais fraco capaz de promover percepção auditiva possui, em média, a intensidade de 0 dBNA. A pressão sonora mínima detectável, no entanto, varia de acordo com a frequência, e tem como referência 20 μPa. O limiar de desconforto para este mesmo som é 120 dBNA e o de dor 140 dBNA. Dependendo da frequência, uma mesma pressão sonora pode, ou não, ser percebida pelo sistema auditivo^(6)^.

Os estímulos sonoros que chegam à orelha são conduzidos por via aérea e via óssea ao sistema auditivo periférico (orelha externa, orelha média, orelha interna e nervo vestibulococlear) onde ocorre a captação do som, energia mecânica, e sua posterior transdução em impulsos nervosos. As fibras nervosas que saem da cóclea (orelha interna) chegam ao sistema auditivo central (vias auditivas do tronco encefálico, tálamo e córtex auditivo) onde o som é compreendido^(7)^.

As ondas sonoras que chegam à orelha externa seguem pelo conduto auditivo externo e provocam vibrações no tímpano. Os ossículos da orelha média transmitem essas vibrações até a janela oval da cóclea. Na cóclea, especificamente no órgão de Corti, as células ciliadas externas (CCE), em contato com a membrana tectória, movimentam-se tonotopicamente, a depender da frequência sonora^(8,9)^.

A movimentação das CCE amplifica o movimento da membrana basilar em sua respectiva frequência de vibração. A amplificação provoca o contato com as células ciliadas internas (CCI) com a membrana tectória e, finalmente, ocorre a transdução mecanoelétrica. A estimulação dos cíclicos das CCI abre os canais iônicos de potássio provocando a despolarização das células, a liberação de neurotransmissores e a codificação das informações acústicas em impulsos elétricos que chegam ao sistema nervoso central por meio do nervo acústico, ramo do nervo vestibulococlear^(8,9)^.

### Processamento sonoro no sistema auditivo

O sistema nervoso central desempenha um papel fundamental em diversas funções, como memória, atenção, linguagem e outras. O sistema auditivo compartilha estruturas e processos neuroanatômicos com outros sistemas. A forma como o sistema auditivo central processa sons verbais e não verbais se modifica. Diante disso, diferentes tipos de estímulos podem gerar diferentes padrões de respostas^(10)^.

A compreensão da fala no sistema auditivo é um processo complexo que envolve fatores internos e externos, como as características acústicas do som e a presença do ruído^(11)^. A fala é um sinal acústico complexo, caracterizado por uma variedade de propriedades, incluindo a presença de harmônicos, variações de amplitude e mudanças rápidas no espectro de frequência^(12)^. Dessa forma, o indivíduo deve ser capaz de detectar as mudanças rápidas de padrões sonoros^(13)^.

A compreensão da fala depende da integridade das estruturas e conexões responsáveis pela codificação dos sons, ou seja, a representação das suas características temporais e espectrais. A transdução do som em impulso elétrico na cóclea e o processamento do som ao longo das vias auditivas são essenciais para a percepção dos sons. Por isso, qualquer comprometimento nesses processos pode prejudicar a compreensão da fala^(14)^.

Essa capacidade de compreensão auditiva é definida como processamento auditivo central (PAC) que corresponde à eficiência com que o sistema nervoso central utiliza as informações auditivas. Para isso, é necessário um conjunto de competências e habilidades auditivas que são responsáveis pela capacidade de localizar, discriminar, reconhecer, armazenar e compreender informações auditivas^(15)^. As alterações do PAC são causadas por problemas no sistema auditivo central. Essas alterações podem ocorrer independentemente de problemas cognitivos e da linguagem^(10)^.

### Avaliação do processamento do som

Existe uma variedade de testes comportamentais que avaliam diferentes habilidades do processamento auditivo, incluindo discriminação auditiva, processamento temporal, escuta dicótica, reconhecimento de fala de baixa redundância, e interação binaural^(16)^.

Além das avaliações audiológicas básicas, como audiometria tonal, audiometria vocal, Medidas de imitância acústica e emissões otoacústicas, os testes eletrofisiológicos podem fornecer informações valiosas sobre a via auditiva e seus processos até o córtex auditivo^(16)^.

A academia americana de audiologia (AAA) recomenda a utilização dos PEA para a avaliação do PAC, visto que estes refletem mecanismos fundamentais do processamento auditivo como codificação, decodificação, discriminação, consciência auditiva e acesso à memória auditiva. Além disso, documenta a influência da maturação e da intervenção no PAC, podendo ser especialmente úteis para comparações intra-sujeitos^(15)^.

Ao registar os PEA em resposta à sílaba /ba/ (Figura 1), por exemplo, é possível avaliar como o sistema auditivo processa as suas características físico-acústicas, incluindo a detecção de mudanças na frequência, forma de onda e duração do som. Essas análises ajudam a entender melhor a codificação neural da fala e podem fornecer informações importantes sobre a função auditiva em diferentes contextos clínicos^(17)^.

### Microfonismo coclear

O microfonismo coclear (MC) é um potencial gerado principalmente a partir das CCE da cóclea. O MC corresponde a uma atividade elétrica que aparece antes das sinapses das células ciliadas com o nervo auditivo, sendo possível visualizar seu registro antes da onda I, no registro do potencial evocado auditivo de tronco encefálico (PEATE), e sua ausência é compatível com alteração na função dessas células^(18)^.

O MC pode ser registrado por meio da eletrococleografia ou do PEATE, sendo necessário realizar a inversão das polaridades do estímulo acústico utilizado para verificar a inversão do registro a fim de confirmar o MC no PEATE^(18)^. A Figura 1 mostra o registro do MC antes da onda I do PEATE, evidenciando a sua contribuição para a codificação sonora.

Essa resposta inicial é crucial, pois estabelece a base para os estágios posteriores do processamento auditivo. Ao preservar a fidelidade do sinal acústico, o MC garante que as informações essenciais, como frequência, intensidade e timing, sejam transmitidas de forma precisa para as estruturas neurais subsequentes. Essa característica torna o microfonismo coclear uma ferramenta valiosa tanto na pesquisa quanto na prática clínica, permitindo a detecção precoce de disfunções auditivas e contribuindo para o desenvolvimento de estratégias diagnósticas e terapêuticas^(19)^.

### Potencial evocado auditivo de tronco encefálico

O PEATE é um PEA de curta latência que ocorre nos primeiros 10 milissegundos (ms) após a apresentação do estímulo acústico e se origina do nervo auditivo e das vias auditivas no tronco cerebral, ou seja, estruturas envolvidas na codificação sonora (Figura 1). O PEATE permite avaliar objetivamente a resposta dos indivíduos em diferentes intensidades, sendo amplamente utilizado para avaliar a integridade das vias auditivas até o tronco encefálico e estimar o limiar auditivo eletrofisiológico. Sua análise consiste na identificação de três picos de onda principais (I, III e V), assim como a reprodutibilidade do traçado, as latências absolutas, as amplitudes, os intervalos interpicos e a diferença interaural das ondas^(20,21)^.

No PEATE, são comumente utilizados estímulos breves, como clique, *tone burst* e *chirp*. Os picos I, III e V são as mais analisadas na prática clínica por possuírem maior estabilidade e amplitude, sendo originadas, respectivamente, na porção distal do nervo auditivo, nos núcleos cocleares e no lemnisco lateral. No entanto, os estímulos breves utilizados no PEATE possuem um padrão acústico simples e diferente dos sons encontrados no ambiente, como os sons da fala, tornando esse PEA limitado para avaliação do comportamento do tronco encefálico, principalmente quando se considera o processamento dos sons da fala nessas estruturas^(20-22)^.

Finalmente, os PEATE capturam a codificação sonora inicial ao registrar, com alta precisão temporal, as respostas elétricas geradas pelo nervo auditivo e pelas vias do tronco encefálico nos primeiros 10 ms após o estímulo, refletindo a sincronização neural essencial para a discriminação de intervalos temporais mínimos e a diferenciação de frequências. Essa capacidade de registrar os picos de onda I, III e V, que representam distintos marcos da transmissão neural, permite avaliar como o sistema auditivo processa e preserva as características temporais e espectrais do sinal acústico, fundamentais para a percepção de sons complexos, como a fala, aprofundada a seguir. Assim, o PEATE não só verifica a integridade estrutural das vias auditivas iniciais, mas também oferece uma janela valiosa para o estudo dos mecanismos de processamento temporal e de frequência, servindo de ferramenta importante tanto na prática clínica quanto em pesquisas sobre distúrbios auditivos^(23)^.

### Frequency following response

O *frequency following response* (FFR) é um índice não invasivo da fidelidade da codificação do som no cérebro e é usado para estudar a integridade, plasticidade e relevância comportamental da codificação neural do som (Figura 1)^(24)^. Esse PEA é realizado com estímulo de fala, sendo a sílaba /da/ utilizada com mais frequência, o que o distingue de outros PEA por refletir o processamento neural das características acústicas de um som e a capacidade de codificação da fala^(24,25)^.

O FFR pode ser interpretado no domínio do tempo, no qual são identificados os picos de resposta na forma de onda, são elas: V, A, C, D, E, F e O. Essas ondas têm como prováveis sítios geradores o tronco encefálico rostral, mais especificamente o lemnisco lateral e o colículo inferior, além do córtex primário^(26)^. Além disso, ao aplicar uma transformada rápida de fourier (FFT), pode ser realizada a análise no domínio das frequências, como a frequência fundamental (F_0_), o primeiro formante (F_1_) e os harmônicos altos (HA). As respostas do FFR são geradas predominantemente no mesencéfalo auditivo, um centro de atividade aferente e eferente, por isso, refletem uma série de influências da via auditiva periférica e do sistema nervoso central^(24)^.

Assim, o FFR pode ser inserido na bateria de exames audiológicos e possui um papel importante no *crosschecking.* Seus resultados promissores na clínica demonstram potencial para auxiliar indivíduos de diferentes faixas etárias, desde bebês até idosos, com diversas necessidades, como dificuldades de aprendizagem, transtorno do déficit de atenção e hiperatividade (TDAH), e transtorno do processamento auditivo^(27)^.

Por fim, O FFR se destaca na análise contínua e precisa das características acústicas essenciais à percepção da fala, pois sua capacidade de capturar os harmônicos e formantes fornece uma representação neural detalhada que reflete as nuances espectrais do estímulo sonoro. Essa fidelidade na preservação do sinal permite a identificação de variações sutis no F0 e na estrutura dos formantes, elementos críticos para a distinção de fonemas e para a compreensão da prosódia. Estudos indicam que uma codificação neural comprometida desses componentes pode estar associada a dificuldades na percepção da fala, sobretudo em ambientes ruidosos, reforçando a relevância do FFR na investigação dos mecanismos subcorticais de integração acústica^(28,29)^. Dessa forma, o FFR não só complementa a avaliação audiológica tradicional, mas também se configura como uma ferramenta promissora para aprimorar estratégias diagnósticas e terapêuticas em transtornos do processamento auditivo.

### Binaural Interaction component

O BIC (*Binaural Interaction Component*) é uma medida eletrofisiológica que avalia a interação entre as vias auditivas de ambos os hemisférios cerebrais. Apesar de poder ser avaliado em diferentes potenciais evocados auditivos, é frequentemente utilizado na avaliação de respostas eletrofisiológicas auditivas de curta latência e de média latência. Ele é obtido subtraindo as respostas evocadas por estímulos monaurais da resposta evocada por estímulos binaurais, refletindo a eficiência da integração de informações sonoras nas vias auditivas centrais^(30)^. O BIC pode ser calculado a partir da seguinte equação genérica:


$$\begin{array}{c} BIC=\;Resposta_{binaural}-\\ \left(Resposta_{monaural\;esquerdo}+Resposta_{monaural\;direito}\right) \end{array}$$ (1)

Essa medida fornece informações sobre a integridade das conexões neurais responsáveis pela audição espacial e pelo processamento de sinais em ambientes complexos, como aqueles com presença de ruído de fundo^(31)^.

O processamento binaural comportamental, por outro lado, refere-se à capacidade de combinar e distinguir sons que chegam aos dois ouvidos, sendo fundamental para habilidades como localização sonora, percepção de profundidade auditiva e compreensão de fala em ambientes ruidosos^(30)^.

Estudos demonstram que há uma correlação significativa entre o BIC e medidas comportamentais de processamento binaural, como a diferença de nível de mascaramento (MLD) e o reconhecimento de dígitos dicóticos^(32,33)^. Isso indica que a capacidade de interação binaural, capturada pelo BIC, está intimamente associada ao desempenho comportamental em tarefas auditivas. Além disso, ambas as medidas são influenciadas igualmente por variáveis como idade e simetria auditiva^(30-33)^. Em especial, indivíduos mais velhos podem apresentar um BIC reduzido e um pior desempenho em testes comportamentais, o que pode ser um indicativo de déficit no processamento auditivo central^(31)^.

### Potencial evocado auditivo cortical

O potencial evocado auditivo cortical (PEAC) é um PEA de longa latência que ocorre entre 50 e 300 ms e é representado pelo complexo P1-N1-P2 que reflete o processamento do som subjacente às habilidades de codificação, decodificação e discriminação auditiva (Figura 1). A decodificação corresponde à interpretação das características temporais e espectrais de um som captadas na codificação, enquanto a discriminação corresponde à capacidade de detectar, reconhecer e distinguir diferenças entre os sons^(34,35)^.

O componente P1 é oriundo das projeções talâmicas e do córtex auditivo primário, possuindo relação com a codificação auditiva^(36)^. O componente N1 é originado das ativações do córtex auditivo primário na região lateral do giro temporal, recebendo também influências do lobo temporal lateral, córtex motor e pré-motor frontal. Esse componente está associado à atenção e à decodificação auditiva^(37)^.O componente P2, relacionado à discriminação auditiva, resulta da ativação conjunta do córtex auditivo primário e de áreas corticais superiores, como as regiões supratemporal, frontal e parietal. Essa ativação complexa permite a análise das características acústicas e temporais do estímulo^(36)^.

Dessa forma, a progressão dos componentes do PEAC – P1, N1 e P2 – evidencia o percurso refinado do processamento sonoro rumo ao córtex, refletindo estágios sucessivos de integração e interpretação de informações acústicas complexas. Enquanto o P1 marca a codificação inicial dos sinais, o N1 representa uma fase intermediária na qual os aspectos temporais e espectrais são integrados, e o P2 denota a interpretação mais elaborada dos elementos acústicos, como harmônicos e formantes, essenciais para a percepção da fala. Essa sequência funcional não só ressalta a maturação e a plasticidade do sistema auditivo central, mas também enfatiza a relevância clínica do PEAC para identificar e monitorar transtornos no processamento auditivo^(38,39)^.

As respostas do PEAC não dependem da participação ativa do indivíduo examinado, ou seja, é uma resposta exógena que pode ser eliciada a partir de diferentes estímulos acústicos, desde tons puros até estímulos de fala. A utilização de estímulos de fala, como a sílaba /ba/ representada na Figura 1, permite avaliar a capacidade do sistema auditivo em processar e discriminar as complexidades acústicas que esses estímulos possuem^(34-37)^.

O PEAC possui grande contribuição na avaliação do PAC, demonstrando sua utilidade na avaliação objetiva do processamento sonoro até o nível do sistema nervoso auditivo central. Em crianças pequenas, o componente P1 é melhor visualizado no traçado e tem sido considerado um biomarcador neurofisiológico do desenvolvimento auditivo à medida que diminui a latência e a amplitude com o passar dos anos^(34,39)^.

### Mismatch negativity

O *Mismatch negativity* (MMN) é um PEA com resposta de longa latência que ocorre por volta de 100 a 350 ms. Seus prováveis sítios gerados são o plano supratemporal do córtex auditivo, córtex temporal posterior lateral, giro frontal direito e contribuições do tálamo. Sua resposta é obtida a partir dos traçados do PEAC, a partir da seguinte equação:


$$Traçado_{MMN}=Traçado_{Raro}-\;Traçado_{Frequente}$$ (2)

As componentes de onda N1 e P2 do PEAC estão diretamente relacionadas ao MMN. O N100, frequentemente utilizado como referência, precede o MMN, que se manifesta como um vale negativo após ele. O P200, por sua vez, associado a processos cognitivos mais complexos, pode fornecer informações adicionais sobre a detecção da mudança detectada pelo MMN^(40)^.

O MMN é um indicador do estágio inicial do processamento auditivo, podendo fornecer medidas objetivas sobre a discriminação auditiva e o processamento automático da percepção do som por ser registrado, mesmo na ausência de atenção consciente ao estímulo sonoro (Figura 1). Isso sugere que o cérebro está continuamente processando informações auditivas em um nível pré-consciente^(40)^.

Por fim, o MMN reflete a capacidade do sistema neural de antecipar e detectar discrepâncias em informações acústicas, servindo como marcador sensível da integridade e plasticidade das vias auditivas. Essa habilidade está intimamente ligada ao processamento auditivo central, onde circuitos neurais complexos integram e interpretam os sinais sonoros para construir percepções significativas. Por isso, o MMN não apenas evidencia a funcionalidade das vias auditivas, mas a capacidade do cérebro de discriminar sons armazenados na memória^(4,41)^.

### Potencial evocado auditivo cognitivo

O Potencial evocado auditivo cognitivo ou P300 é considerado um PEA de longa latência gerado a partir de respostas do córtex auditivo, lobo frontal, hipocampo e sistemas sensoriais. Trata-se de um PEA endógeno, ou seja, é gerado voluntariamente, pois requer uma tarefa do indivíduo enquanto os estímulos acústicos são apresentados^(35,42)^.

Este PEA possui um pico positivo obtido através da identificação de um estímulo raro em meio a uma série de estímulos frequentes apresentados (*oddball paradigm*), a partir da realização de uma tarefa discriminatória específica. O componente P300 aparece por volta de 300 ms, sendo o maior pico positivo após o complexo N1-P2 (Figura 1).

Assim, o P300, como um PEA endógeno gerado por respostas cognitivas do sistema auditivo, está intimamente conectado ao processamento auditivo central, uma vez que reflete a integração de informações acústicas com funções cognitivas superiores, como atenção e tomada de decisão. A habilidade do cérebro em identificar estímulos raros revela a eficiência das redes neurais envolvidas na discriminação auditiva e no processamento sequencial da informação, com a participação ativa das áreas corticais auditivas, parietais e do hipocampo. Essa capacidade de modulação cognitiva das respostas auditivas reforça a importância do P300 na avaliação da interação entre os processos centrais de percepção auditiva e os sistemas cognitivos, oferecendo valiosos insights sobre a integridade do processamento auditivo central e suas implicações para a atenção e memória auditiva^(5,43)^.

Então, a partir das respostas do P300 é possível avaliar habilidades de cognição, atenção, discriminação, memória, tomada de decisão e processamento sequencial da informação auditiva^(34,35,42)^.

### N400

O N400 é um potencial cognitivo negativo de longa latência, eliciado por uma palavra semanticamente incongruente ou inesperada, apresentada entre outras palavras as quais guardam conexões semânticas. O componente N400 é um pico negativo encontrado por volta de 400 ms responsável por captar informações sobre aspectos psicolinguísticos que não são avaliados em nenhuma outra abordagem ou avaliação comportamental e/ou auditiva^(43)^.

Desse modo, esse PEA é considerado um marcador neurofisiológico da discriminação de sons/palavras e de sua relação de congruência semântica de um estímulo linguístico. A geração do componente N400 requer atenção auditiva e consciência, sendo capaz de refletir a compreensão baseada no processamento sensorial e associação do significado^(44)^.

## CONCLUSÃO

O modelo proposto, fundamentado na análise dos PEA, oferece uma visão integrada e funcional do processamento auditivo, traçando uma narrativa coerente que abrange desde a captação das características acústicas iniciais até a interpretação cortical dos estímulos sonoros. Cada PEA discutido – desde o microfonismo coclear, PEATE e o FFR, que evidenciam a codificação e a fidelidade dos sinais na entrada neural, até BIC, PEAC, MMN, P300 e N400, que refletem, a integração, codificação, decodificação, discriminação e resposta cognitiva aos estímulos – contribui para a compreensão dos estágios sucessivos do processamento sonoro. Essa abordagem abrangente não só permite uma compreensão mais ampla sobre diagnóstico e o monitoramento de distúrbios auditivos, como também fundamenta intervenções terapêuticas direcionadas, demonstrando a relevância prática e clínica do modelo na avaliação da integridade, plasticidade das vias auditivas e do funcionamento geral do sistema auditivo.
